# A multi-objective decision-making approach to the journal submission problem

**DOI:** 10.1371/journal.pone.0178874

**Published:** 2017-06-05

**Authors:** Tony E. Wong, Vivek Srikrishnan, David Hadka, Klaus Keller

**Affiliations:** 1 Earth and Environmental Systems Institute, Pennsylvania State University, University Park, Pennsylvania, United States of America; 2 Department of Energy and Mineral Engineering, Pennsylvania State University, University Park, Pennsylvania, United States of America; 3 Department of Computer Science and Engineering, Pennsylvania State University, University Park, Pennsylvania, United States of America; 4 Department of Geosciences, Pennsylvania State University, University Park, Pennsylvania, United States of America; 5 Department of Engineering and Public Policy, Carnegie Mellon University, Pittsburgh, Pennsylvania, United States of America; Centre for Research and Technology-Hellas, GREECE

## Abstract

When researchers complete a manuscript, they need to choose a journal to which they will submit the study. This decision requires to navigate trade-offs between multiple objectives. One objective is to share the new knowledge as widely as possible. Citation counts can serve as a proxy to quantify this objective. A second objective is to minimize the time commitment put into sharing the research, which may be estimated by the total time from initial submission to final decision. A third objective is to minimize the number of rejections and resubmissions. Thus, researchers often consider the trade-offs between the objectives of (i) maximizing citations, (ii) minimizing time-to-decision, and (iii) minimizing the number of resubmissions. To complicate matters further, this is a decision with multiple, potentially conflicting, decision-maker rationalities. Co-authors might have different preferences, for example about publishing fast versus maximizing citations. These diverging preferences can lead to conflicting trade-offs between objectives. Here, we apply a multi-objective decision analytical framework to identify the Pareto-front between these objectives and determine the set of journal submission pathways that balance these objectives for three stages of a researcher’s career. We find multiple strategies that researchers might pursue, depending on how they value minimizing risk and effort relative to maximizing citations. The sequences that maximize expected citations within each strategy are generally similar, regardless of time horizon. We find that the “conditional impact factor”—impact factor times acceptance rate—is a suitable heuristic method for ranking journals, to strike a balance between minimizing effort objectives and maximizing citation count. Finally, we examine potential co-author tension resulting from differing rationalities by mapping out each researcher’s preferred Pareto front and identifying compromise submission strategies. The explicit representation of trade-offs, especially when multiple decision-makers (co-authors) have different preferences, facilitates negotiations and can support the decision process.

## Introduction

Researchers often face the decision problem of where to submit a manuscript. In addition to potential professional benefits, the quick publication of papers where they are likely to be widely read improves the public good obtained by the generation and dissemination of scientific knowledge.

Some authors and institutions focus on the impact factor of the journals in which a researcher publishes as a measure of the reach of the researcher’s work [1,2]. This is motivated by the fact that a journal’s impact factor is intended to correlate with the average number of times per year a paper in a given journal is cited [3]. The use of impact factors is often criticized, however. For example, impact factor is a journal-level metric, not author-level, so their usefulness for evaluating the productivity of an individual researcher is limited [2,4]. Additionally, impact factors fail to take into account numerous non-citation considerations in determining an author’s scientific reach (such as social media activity, media interest, page and article views, influence in policy documents and general interest outside the core scientific community) [4,5]. Impact factors also tend to overemphasize a small number of highly-cited papers [6] and they do not compare well across disciplines due to different field characteristics such as size and publication rates [1,6–9]. Nevertheless, impact factors are often viewed as a rough estimate of the expected number of times an article in a given journal will be cited in other peer-reviewed work in a one-year period. We—noting the caveats listed above—follow the example set by previous work and use impact factor as an estimate of how influential a paper is likely to be when published in a particular journal [10]. We discuss this assumption further in Methods.

However, researchers may not only select journals to maximize the potential impact of a publication. Researchers may also seek to minimize the time length of the review process (as well as the frustrations and extra time commitments stemming from multiple revisions and resubmissions), helping to ensure that new knowledge reaches its audience as quickly as possible. The “journal submission decision” problem was introduced by ref. [11] and more recently formalized and expanded along these lines [10]. In this framework, the researcher(s) must select a sequence of journals to which to submit the manuscript. This submission decision problem features multiple objectives, including: (i) the expected number of citations the article to-be-submitted will accumulate over a given time horizon (ii) the expected number of (rejections and) resubmissions; and (iii) the expected length of time from submission to acceptance [10]. The submission decision problem was expanded by ref. [12], who incorporated a representation of the uncertainties and complications inherent in the process of ranking journals beyond impact factor.

Complex decision problems often feature *deep*, or *Knightian*, *uncertainties*, in which decision-makers cannot agree on the set of possible outcomes, the consequences of those outcomes, and/or the associated probability distributions [13–15], as well as *multiple decision-maker rationalities* [16]. For example, the trade-offs between objectives for co-authors on the same manuscript may not always be aligned due to the co-authors’ different career stages. The co-authors may also have conflicting prior assumptions regarding the probability that their manuscript will be “scooped” (that is, a journal article of similar scope is published prior to their manuscript being accepted for publication, potentially reducing the novelty and impact of the manuscript). This perceived “scooping” probability is an example of a deeply uncertain parameter even for the single-author case, as it is mostly based on (often diverging) subjective estimates. Furthermore, differing objectives and prior assumptions between authors illustrate the importance and difficulty of balancing multiple decision-maker rationalities. Previous analyses of the submission decision problem have illustrated the development of optimal submission strategies for a single researcher, but has been silent on the issues of (i) finding strategies to compromise between the interests of multiple decision-makers and (ii) the impacts of deeply uncertain factors in the decision problem [10,12,17].

Here, we expand upon previous studies and offers a formal method to navigate the concerns that result from deep uncertainty and multiple decision-maker rationalities. We employ a Many-Objective Robust Decision-Making (MORDM) approach, using a set of tools designed to inform decision-making through transparency [18]. Decision-making in cases of deep uncertainty can be informed by explicit and transparent representation of the trade-offs. This representation can then help to identify solutions that are acceptable to the different decision-makers that often have diverging preferences [19]. We evaluate the impacts of deeply uncertain factors (e.g., perceived scooping probability). We focus on introducing the MORDM methodology to the journal submission decision problem and demonstrating how to discover and navigate the tensions brought on by deep uncertainty. A full Many-Objective *Robust* Decision-Making approach would include a “stress test” of the proposed submission strategies to discover scenarios in which the strategies might perform poorly. This is beyond the scope of this study, although we note that recent work has proposed and demonstrated a framework for endogenizing the “scenario discovery” within the MORDM approach [20]. We explicitly represent the trade-offs inherent in the submission decision process, and illustrate the impacts of deep uncertainties such as contrasting co-author views of the probability of their manuscript being scooped and the length of time needed to make revisions. This quantitative formulation of the journal submission decision problem, both in the present work and as it was originally formulated [10], is accompanied by many caveats, and should not be taken as a blanket endorsement of one journal or set of journals or a prescription of a particular submission sequence. However, we defer additional discussion of these caveats until the section Discussion and Caveats.

## Methods

### Representing the journal submission decision problem

We adopt as a starting point the insightful model of the journal submission decision problem described in ref. [10]. In this model, a researcher has finished a manuscript and must decide to which journal to submit. This decision, when viewed as an optimization problem, can have many objectives. We focus here on three objectives: (i) maximize the expected number of citations the paper will accrue over a fixed time period; (ii) minimize the expected number of times the manuscript will be rejected (and consequently revised and resubmitted); and (iii) minimize the expected amount of time from initial submission to final acceptance (or rejection, in the event that the manuscript has been rejected from all journals to which it was submitted). The following problem formulation is based on ref. [10], with several modifications described below. We provide an overview of the problem set-up and key equations. A detailed mathematical construction of the model is provided in greater detail in Supplementary Text.

Let *T* represent the time horizon over which the researcher wishes to maximize citations. *T* might be the duration of a postdoctoral appointment (say, three years) for a junior researcher who is interested in applying for tenure-track jobs. Alternatively, *T* could represent the time until a final decision is made regarding tenure (say, seven years), or the projected remainder of a tenured researcher’s career (say, 20 years).

The impact factor (IF) of a journal is calculated as

$$IF=\frac{Q}{M},$$ (1)

where *Q* is the number of citations in the current year to items published in the journal the previous two years and *M* is the number of items published in the journal during the same two years [3]. Let *q*_*i*_ represent the number of citations in the current year to items published in the previous two years for publication *i =* 1, 2, …, *M* in the journal in question, where *i* enumerates the items published in the journal over the previous two years. Then Eq (1) becomes

$$IF=\frac{1}{M}\sum_{i=1}^{M}q_{i},$$ (2)

which is the mean number of citations in the current year for an item published in the journal during the past two years. In light of the lack of journal-specific data regarding a probability distribution for the number of citations per article per year, we use the impact factor as a point estimate for the expected value (in the mean sense) of the number of citations per year for an article published in that journal. Furthermore, we make the assumption that the impact factor remains constant over the time period considered, which is almost certainly not the case in reality.

Let an enumeration of journals be indexed by *j*, and suppose the authors of a manuscript only intend to submit to *N* journals total. Suppose the authors intend to submit the manuscript, in order, to journal *j =* 1, 2, …, *N*. Let α_*j*_, λ_*j*_, and τ_*j*_ denote the acceptance rate, expected number of citations for an article in the journal over the course of a year, and the expected time from submission to publication for journal *j*, respectively. Let *s* denote the probability (per day) of an article being scooped and *t*_*R*_ denote the time (in days) required to make revisions and resubmit a manuscript. Following previous work, we adopt the assumption that a manuscript that has been “scooped” is essentially worthless and receives no citations, and that the probability of being scooped is constant in time [10]. As in previous work [10], the perceived probability of scooping (*s*) is a function of the preferences and perceptions of the individual decision-makers. Different fields, research topics, and decision-maker perceptions will suggest higher or lower values for s are appropriate for different applications. Decision-makers may want to examine the cumulative probability of scooping, (1-*s*)^*t*^ (where *t* is time in days), to decide what values of *s* are realistic for their application. The total probability of a manuscript being rejected from all *N* journals in a submission sequence is

$$p_{r}=1-\sum_{j=1}^{N}\alpha_{j}\prod_{k=1}^{j-1}(1-\alpha_{k}){\left(1-s\right)}^{\tau_{k}+t_{R}}.$$ (3)

Thus, the total probability of acceptance at a journal in the submission sequence is 1-*p*_*r*_. We include this in the results as a quantity to help navigate trade-offs, but do not include it explicitly as an objective because the acceptance rate for each journal is included in the other objectives. The expected number of citations for the manuscript is then

$$C=\sum_{j=1}^{N}\alpha_{j}\lambda_{j}{\left[T-(\sum_{i=1}^{j}\tau_{i})-(j-1)t_{R}\right]}^{+}\prod_{k=1}^{j-1}(1-\alpha_{k}){\left(1-s\right)}^{\tau_{k}+t_{R}}+p_{r}*0,$$ (4)

where the superscript + on the term in square brackets in Eq (4) implies that negative values should be replaced with zero. The *p*_r_*0 term in Eq (4) accounts for the fact that a manuscript which has been rejected *N* times earns zero citations. In practice, authors may submit such a manuscript to journals with subsequently higher acceptance rates to reduce the probability of overall rejection, rather than worrying about citations.

There are several simplifying assumptions in the formulation of Eq (4). First, the impact factor of each journal is assumed to be constant over the time horizon. Second, the “impact” of a paper is time-invariant: the rate of citations does not change with time. Finally, as a consequence of the available data, we assume that the time a paper is under review at a particular journal is precisely the mean time for that journal.

The expected total number of submissions of a manuscript is

$$\begin{array}{l} R=\sum_{j=1}^{j_{max}}j\;\alpha_{j}\prod_{k=1}^{j-1}\left[(1-\alpha_{k}){\left(1-s\right)}^{\tau_{k}+t_{R}}\right]\\ \;+j_{max}\left(1-\sum_{j=1}^{j_{max}}\alpha_{j}\prod_{k=1}^{j-1}\left[(1-\alpha_{k}){\left(1-s\right)}^{\tau_{k}+t_{R}}\right]\right), \end{array}$$ (5)

where *j*_*max*_ is the expected maximum number of submissions within the time horizon *T* along a given submission sequence. That is, *j*_*max*_ is the largest index *j* along the submission sequence such that the following expression is true.

$$T-\sum_{i=1}^{j}\tau_{i}-(j-1)t_{R}>0$$ (6)

The formulation of Eq (5) differs from that in ref. [10], in that we limit the maximum number of submissions based on the expected revision and review time of the proposed journal submission sequence instead of using a Heaviside function to assign zero probability to submission numbers exceeding the time limit.

The expected total time spent under review along a given journal submission sequence is

$$\begin{array}{l} P=\sum_{j=1}^{j_{max}}\alpha_{j}(\sum_{i=1}^{j}\tau_{i}+(j-1)t_{R})\prod_{k=1}^{j-1}\left[(1-\alpha_{k}){\left(1-s\right)}^{\tau_{k}+t_{R}}\right]\\ \;+min\left(T,\sum_{i=1}^{j_{max}}\tau_{i}+(j_{max}-1)t_{R}\right)\left(1-\sum_{j=1}^{j_{max}}\alpha_{j}\prod_{k=1}^{j-1}\left[(1-\alpha_{k}){\left(1-s\right)}^{\tau_{k}+t_{R}}\right]\right). \end{array}$$ (7)

This can be interpreted as the expected time until the final decision, which occurs at one of three points: (i) acceptance by one of the *N* journals, (ii) rejection by all *N* journals, or (iii) expiration of the time horizon before the manuscript could be accepted. This time includes the manuscript revision time *t*_*R*_ between each resubmission. If the time horizon *T* expires before the review process can be completed at all *N* journals along the submission sequence, then the final decision occurs at time *T*. The parameters *s*, *t*_*R*_, and *T* are considered uncertain factors that depend on the perspectives and preferences of the authors of the manuscript. The quantities α_*j*_, λ_*j*_, and τ_*j*_ are properties of the journals to which the manuscript might be submitted.

### Data

The model of Eqs (4), (5) and (7) requires the mean acceptance rate (α_*j*_), impact factor (λ_*j*_), and mean time from submission to final decision (τ_*j*_) for each potential journal the researchers might submit to. We use the data set from ref. [10], which permits a transparent comparison of methodologies from that work and the results presented here. This data set contains the required information for a set of 61 journals that either specialize in Ecology or are general journals that publish ecological research. The data set spans a wide range across the impact factor spectrum and is available from ref. [10] (see S1 Table in [10]).

In the original formulation [10], Eq (4) is normalized to ensure that the probability of being accepted to a particular journal forms a probability distribution. This normalization implicitly assumes that the manuscript will be accepted to one of the journals along the sequence (i.e., *p*_*r*_ = 0). When all 61 journals in the data set are included in each submission sequence, this is a reasonable assumption; the probability of being rejected from all 61 journals is about 2x10^-13^ (neglecting scooping). Here, we consider only journal submission sequences of length *N* = 5. This parameter choice reflects the authors’ experience that many researchers try to limit the number of revisions and resubmissions. As a (convenient) side effect, this choice also keeps the computational demands within a reasonable scale. With this assumption, there is a non-negligible chance that a manuscript will be rejected from all five journals in a given submission sequence.

### XLRM framework

**Fig 1:**
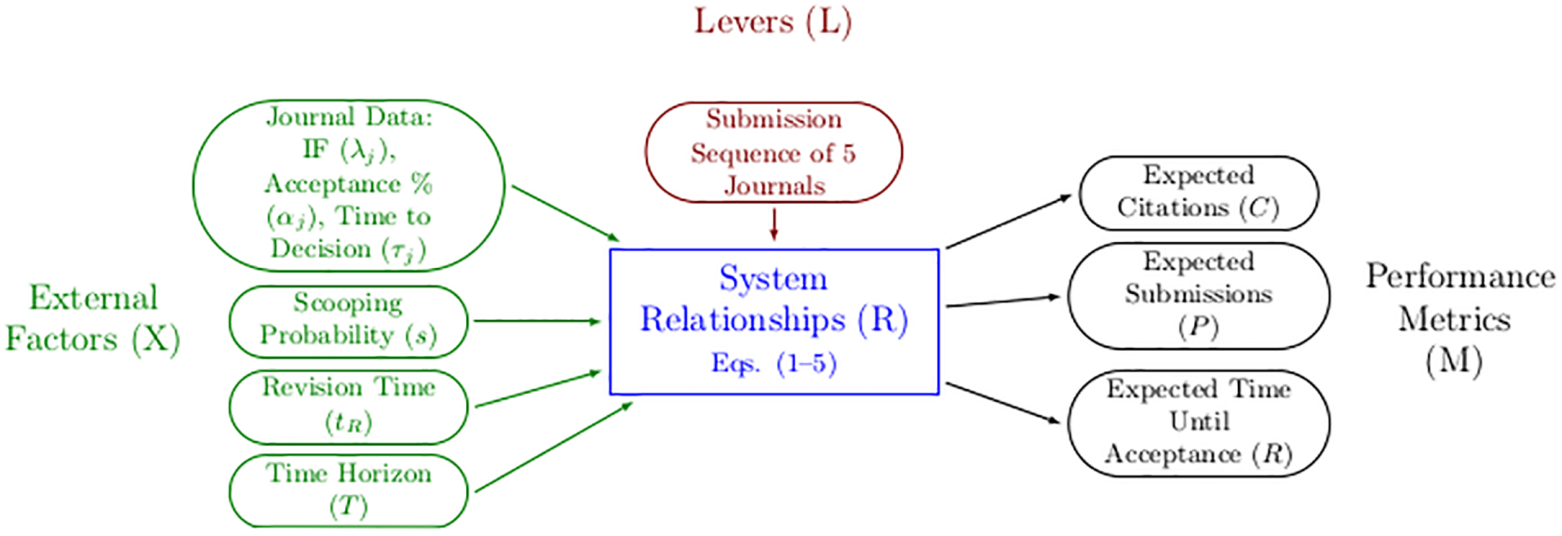
XLRM framework. Illustration of the external factors (X), levers the decision-maker can manipulate to affect the outcome (L), the modeling relationships (R) and the performance metrics (M) in the formulation of the journal submission decision problem [10].

The “XLRM” framework is a simple approach to synthesize the formulation of a decision analysis [21] (Fig 1). This framework casts the decision problem in terms of external factors that the decision-maker must take into account in the decision process (X), levers the decision-maker may manipulate that constitute the actual “decision” (L), the system relationships (R), and the performance metrics on which the optimality of the decision is based (M).

In our formulation of the journal submission decision problem, the external factors consist of the journal-specific data (acceptance rates (α_*j*_), impact factors (λ_*j*_), and time-to-decision (τ_*j*_)), the scooping probability (*s*), the time horizon (*T*) over which the decision-maker wishes to accrue citations, and the time needed to make manuscript revisions (*t*_*R*_). The scooping probability, time needed to make revisions, and time horizon are uncertain factors that may vary from researcher to researcher. The decision lever in this problem is the chosen ordered sequence of *N* journals to which to submit the manuscript. The performance metrics are the three objectives of maximizing citations, minimizing time in review, and minimizing the number of submissions (Eqs 4, 5 and 7, respectively).

### Multi-objective decision-making

Choosing a strategy in the face of multiple objectives often requires the navigation of trade-offs among the objectives. In other words, different solutions may perform better than others with respect to particular objectives. For these types of problems, decision-makers are often interested in characterizing the entire set of solutions that are *non-dominated*—that is, for which no other solution performs better across all objectives (an improvement in one objective requires a deterioration in another) [22]. Non-dominated solutions are also called *Pareto-optimal*, and the set of objective values corresponding to Pareto-optimal solutions is the *Pareto front* [22]. The identification of the Pareto front prior to decision-making allows decision-makers to explore preferred solutions based on an understanding of the relevant trade-offs. This form of optimization is known as *a posteriori* decision-making (as opposed to *a priori* decision-making, in which preferences are specified prior to the optimization process) [23].

For even relatively simple problems, it can be computationally intractable to enumerate all possible solutions to find the “true” Pareto front. In this relatively stylized example, there are over 700 million possible permutations of five journals out of the total set of 61 journals; direct identification of the exact Pareto front would involve simulating all of these permutations and comparing their objective values. While technically still feasible (but arguably cumbersome) in this case, we apply a method to identify high-quality approximations of the Pareto front. One class of these computational methods are Multi-Objective Evolutionary Algorithms (MOEAs) [23,24]. These algorithms utilize a population-based search to identify an approximate Pareto front. For this work, we use the NSGA-II (Non-Dominated Sorting Genetic Algorithm-II) MOEA, which uses the Pareto-dominance relation to search the lever space for Pareto-approximate solutions [25]. We examine the number of function evaluations required by the MOEA employed here as well as the Metropolis algorithm used by ref. [10] in order to discover the approximate Pareto front.

In the face of deep uncertainties, MOEAs can be combined with methods from robust decision-making to form the Multi-Objective Robust Decision-Making (MORDM) framework [18,24,26]. Solutions from the Pareto front are tested against various states of the world to identify regions of the parameter space where the solution performs “poorly,” as defined by the decision-maker. While we do not fully utilize the power of the MORDM framework here, our analysis can be extended to examine how select submission sequences cope with uncertain time horizons, revision times, and scooping probabilities. To illustrate this in a limited fashion, we demonstrate the consequences of multiple decision-maker rationalities, where different decision-makers have different beliefs or preferences. In particular, we consider the case of a collaborative paper between a junior author (for example, a Ph.D. student) and a senior author (say, this Ph.D. student’s advisor). We determine the approximate Pareto front for each co-author based on their preferred time horizon. A more complex approach, which we do not employ here but is an interesting avenue for future study, is to include both researchers’ objectives explicitly in the same multi-objective optimization formulation.

## Results

### Performance comparison

**Fig 2:**
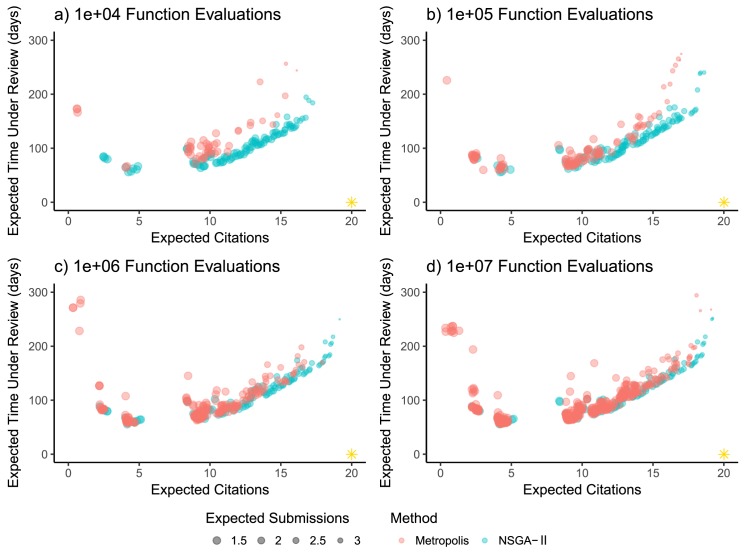
Performance comparison. The approximate Pareto front between expected citations (horizontal axis), expected time under review (vertical axis) and expected number of submissions (size of points), as discovered by the Metropolis algorithm (red points) or NSGA-II MOEA (blue points) for (a) 10^4^, (b) 10^5^, (c) 10^6^ and (d) 10^7^ function evaluations. The model was run with *T* = 3 years, s = 0.001 and *t*_*R*_ = 30 days. The gold star in the lower-right corner represents the target direction for the represented objectives.

First, we compare the approximate Pareto front from the original Metropolis algorithm-based method [10] with the MOEA-based method outlined here in Methods for a varying number of function evaluations (Fig 2). For these experiments, we use a time horizon of *T* = 3 years, a scooping probability *s* = 0.001, and revision time *t*_*R*_ = 30 days. The NSGA-II algorithm finds the basic shape of the approximate Pareto front within 10^4^ function evaluations (Fig 2A). By contrast, the Metropolis algorithm requires about 10^7^ function evaluations to converge to a similar approximate front. Even with 10^7^ function evaluations, several solutions on the Metropolis front are dominated by solutions on the NSGA-II front, suggesting that the NSGA-II algorithm outperforms the Metropolis algorithm in this case.

### Visualization and navigation of multi-objective trade-offs

**Fig 3:**
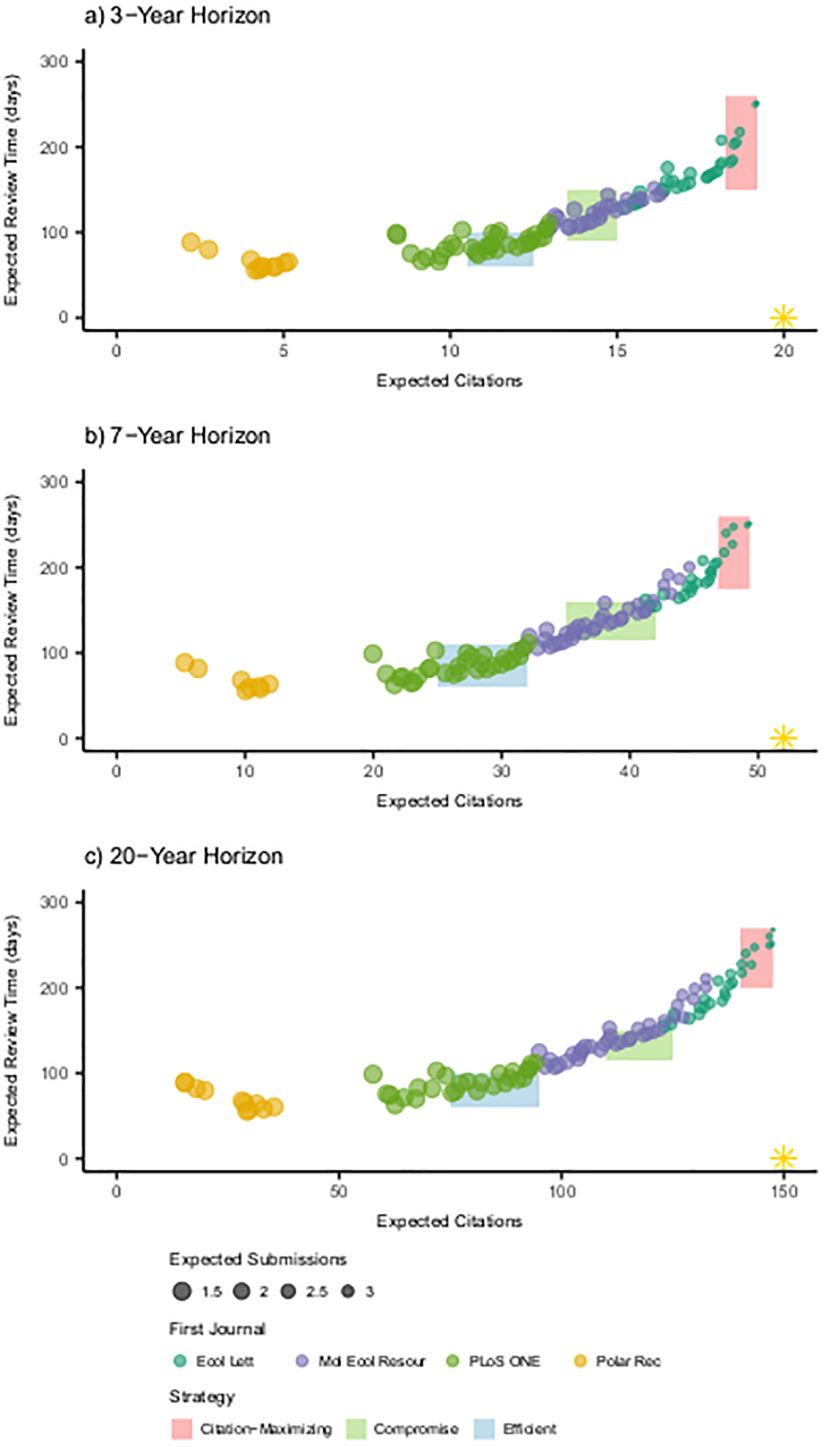
Pareto-approximate solutions. Approximate Pareto fronts for the (a) junior researcher (*T* = 3 years), (b) tenure-seeking researcher (*T* = 7 years) and (c) tenured researcher (*T* = 20 years). The shaded boxes depict the three strategies: Efficient (blue shaded box), Compromise (green shaded box) and Citation-Maximizing (red shaded box). The gold stars in the lower-right corner represent the target direction for the represented objectives.

The overall geometry of the approximate Pareto front is mostly invariant with respect to the time horizon (Fig 3). This is because the expected total time under review along the approximate Pareto front remains between about 50 to 250 days for the 3-, 7- and 20-year time horizons. This leaves more time for citation accumulation than is spent under review, even for the relatively short 3-year horizon. Similarly, the expected number of submissions (Fig 3, dot size) ranges from just over one to under four submissions, also regardless of the time horizon. However, the maximum number of expected citations scales approximately with the number of years in the researcher’s time horizon. The Pareto-approximate sequences appear in clusters roughly organized by the first journal in the sequence. Sequences with low-submission counts, review time, and expected citation sequences typically begin with *Polar Record*. Sequences with high citation counts typically begin with *Ecology Letters*. Finally, submission sequences with moderate citation counts, time in review, and numbers of submissions begin with either *PLOS ONE* or *Molecular Ecology Resources*, depending on the balance between the “effort” metrics of review time and submissions versus the citation count. Note that despite this tendency for the approximate Pareto front to be organized by the first journal, the subsequent journals in each approximately Pareto-optimal submission sequence can vary substantially, illustrating the importance of examining the entire sequence. For these results, we use the MOEA-based approach with 10^7^ function evaluations, scooping probability *s* = 0.001 and revision time *t*_*R*_ = 30 days, unless otherwise stated.

#### Journal submission strategies

We find three distinct regions that represent different strategies a researcher could follow in submitting a manuscript (Fig 3). We select the boundaries between these regions to roughly correspond to the position of the inflection points on the citation count versus time under review Pareto front. The three strategies are: (i) the “Efficient Strategy” (no fewer than 50% of the maximum expected citations and less than 100 days expected under review); (ii) the “Compromise Strategy” (at least 67–70% of the maximum expected citations and no more than 150 days under review); and (iii) the “Citation-Maximizing Strategy” (at least 95% of the maximum expected citations). The shaded boxes in Fig 3A, 3B and 3C depict these strategies for junior (*T* = 3 years), tenure-seeking (*T* = 7 years), and tenured (*T* = 20 years) researchers.

The Efficient Strategy aims to balance the effort the researcher must dedicate to revisions and resubmissions, while pushing the expected citation count as high as possible before the upwards inflection in expected time under review (seen in Fig 3A at about 13 citations). The Efficient Strategy typically requires half the time under review of a Citation-Maximizing Strategy. The Compromise Strategy also avoids the steep increase in expected review time that accompanies a Citation-Maximizing Strategy. The expected number of submissions is less than two for all Pareto-approximate submission sequences following an Efficient Strategy, and less than three for the Compromise Strategy. The increased number of submissions and long review time relative to a 3-year time horizon may make the Citation-Maximizing Strategy less attractive than Efficient or Compromise to the junior researcher, in light of the small number of additional citations to be gained within their time horizon relative to alternative strategies. The longer time horizons of the senior researchers provide the flexibility to pursue higher citation counts, particularly as the potential benefits from more expected citations is larger than for the junior researcher (Table 1). This effect is highlighted by examining expected citations per year for each researcher (expected citation count normalized by the time horizon; Table 1).

**Table 1:** Submission sequences to maximize citations under each strategy.

	Time horizon	Submission sequence	Expected citations	Expected submissions	Expected time under review	Acceptance probability
Citation-Maximizing Strategy	3 years	1. *Ecol Lett* 2. *Science* 3. *Mol Ecol Resour* 4. *Ecol Monogr* 5. *PLOS ONE*	6.4 y^-1^	3.8	251.7 days	0.728
	7 years	1. *Ecol Lett* 2. *Science* 3. *Ecol Monogr* 4. *Mol Ecol Resour* 5. *PLOS ONE*	7.0 y^-1^	3.8	251.7 days	0.728
	20 years	1. *Ecol Lett* 2. *Science* 3. *ISME J* 4. *Mol Ecol Resour* 5. *PLOS ONE*	7.4 y^-1^	3.8	268.0 days	0.716
Compromise Strategy	3 years	1. *Ecol Lett* 2. *Mol Ecol Resour* 3. *PLOS ONE* 4. *Biotropica* 5. *Evol Ecol*	5.5 y^-1^	2.9	149.0 days	0.840
	7 years	1. *Mol Ecol Resour* 2. *Ecol Lett* 3. *Ecol Monogr* 4. *PLOS ONE* 5. *J Evol Biol*	5.9 y^-1^	2.9	149.5 days	0.833
	20 years	1. *Mol Ecol Resour* 2. *Ecol Lett* 3. *Ecol Monogr* 4. *PLOS ONE* 5. *Biotropica*	6.0 y^-1^	2.9	148.1 days	0.841
Efficient Strategy	3 years	1. *PLOS ONE* 2. *Ecol Lett* 3. *Mol Ecol Resour* 4. *ISME J* 5. *Ecol Monogr*	4.3 y^-1^	1.9	94.2 days	0.873
	7 years	1. *PLOS ONE* 2. *Ecol Lett* 3. *Ecol Monogr* 4. *ISME J* 5. *Mol Ecol Resour*	4.5 y^-1^	2.0	95.7 days	0.871
	20 years	1. *PLOS ONE* 2. *Ecol Lett* 3. *Mol Ecol Resour* 4. *ISME J* 5. *Ecol Monogr*	4.6 y^-1^	1.9	94.2 days	0.873

We identify submission sequences for each researcher that maximize the number of citations under each of the three submission strategies (Table 1). These sequences generally feature the same set of journals, though there are some differences in the order in which they appear. It is perhaps surprising that the sequences that maximize citations within each strategy are quite similar across time horizons: aside from the Compromise Strategy, the first two journals in each set of sequences are identical, and the remaining journals are rearranged or are replaced by journals with similar characteristics. The overall Citation-Maximizing Strategies for all three researchers start by submitting to *Ecology Letters*, then to *Science*, and *PLOS ONE* last. That *Ecology Letters* appears before *Science* is likely a result of the higher acceptance rate and faster review time of the former. The Compromise Sequence is also quite similar, though it appears to be more optimal for the researcher with a 3-year horizon to submit to the higher-impact factor *Ecology Letters* before *Molecular Ecology Resources*, while the researchers with longer horizons should submit to *Molecular Ecology Resources* first. Numerically, this is likely related to *PLOS ONE*, with a high acceptance rate and fast review time, being the third journal in the 3-year horizon sequence rather than the fourth. We stress that the sequences presented in Table 1 neglect the effects of varying manuscript quality and content. These considerations may be examined, for example, by reducing the pool of journals to only those where the manuscript is appropriate based on content and quality.

#### Explicit representation of trade-offs

**Fig 4:**
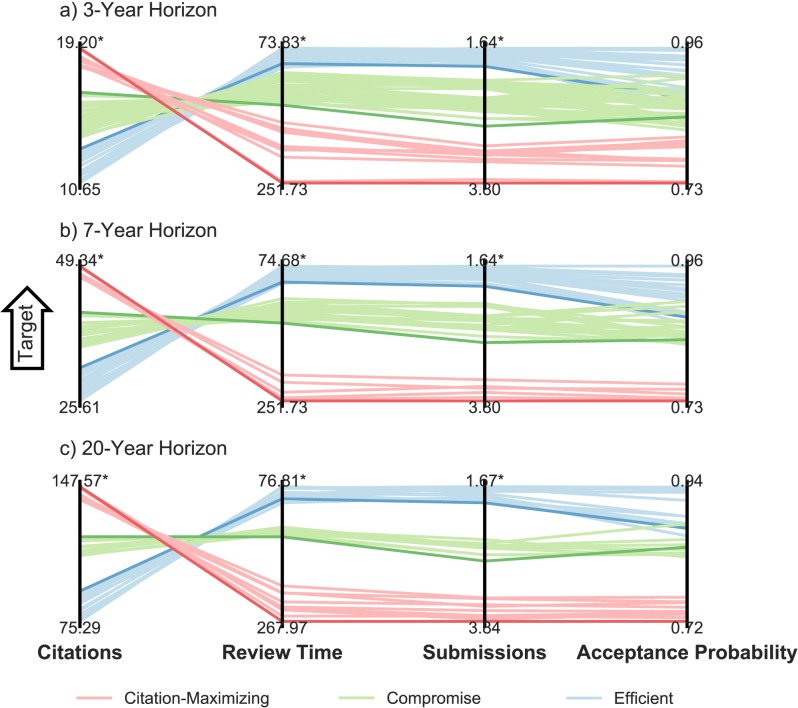
Visualizing multi-objective trade-offs. Parallel axis plots for the (a) junior researcher (*T* = 3 years), (b) tenure-seeking researcher (*T* = 7 years) and (c) tenured researcher (*T* = 20 years). Each vertical axis represents one of the three objectives, with the best simulated values at the top. Each horizontally-oriented shaded region represents a different journal submission strategy and bounds the range of Pareto-approximate solutions for that strategy. Red follows Citation-Maximizing Strategies, blue follows Efficient Strategies (moderate citations and fast publication) and green follows Compromise Strategies. The bold solutions denote the maximum-citation sequence within each strategy.

There are strong trade-offs between the citations objective and each of the two effort objectives, depending on the submission strategy pursued (Fig 4). These trade-offs follow approximately the same shape, where the only difference is that longer researcher time horizons allow for proportionally higher maximum citation counts. In the parallel axis plots shown in Fig 4, each line corresponds to a different journal submission sequence found to be Pareto-approximate for that researcher. Trade-offs between objectives are represented as intersections between solutions from one objective’s axis to the next. This illustrates how a high performance along one objective (near the top of that objective’s axis) might come at the cost of low performance along another objective (near the bottom of the other objective’s axis). In our three-objective formulation of the journal submission decision problem, the three-dimensional approximate Pareto fronts of Fig 3 are readily interpretable. In higher dimensions, however, a graphical representation of all objectives becomes more complex. For example, we might also consider publication costs as an additional objective to minimize, resulting in a four-dimensional Pareto surface.

For all three researchers, Efficient and Citation-Maximizing submission sequences that perform well on expected citations (i.e., near the top of the axis) tend to perform poorly in expected number of submissions and time in review, and vice versa. This demonstrates a strong trade-off between the expected number of submissions (and time in review) and the expected number of citations (Fig 4, central and left-most axes). There is little trade-off between the “effort objectives” of expected total time under review and expected number of submissions (Fig 4, center two axes). As expected, Citation-Maximizing Strategies have the lowest total probability of acceptance, while Efficient Strategies tend to have the highest. That there are some Efficient Strategies with lower total acceptance probabilities than some Compromise Strategies, with no analogous trade-offs on the citation axis, may indicate that those Efficient solutions should be ranked lower than the higher-acceptance probability Compromise solutions, though this depends on the preferences of the researcher with regard to the effort objectives.

#### A heuristic rule to develop a compromise strategy

It may be counterintuitive that *Science*, the journal with the highest impact factor in the data set (31), does not appear as the first submission option for any of the Citation-Maximizing sequences depicted in Figs 3 and 4. Indeed, the strategy of submitting to a high-impact factor journal first, then subsequently lower impact factor journals as a manuscript is rejected, is commonly used in practice [27]. To understand why this strategy might be suboptimal, we order the journals in the data set according to their impact factor multiplied by their acceptance rate (which we call the “conditional impact factor”). Then the top five journals are: *PLOS ONE* (2.82), *Molecular Ecology Resources* (2.60), *Ecology Letters* (2.23), *Ecological Monographs* (2.18), *Science* (2.17). This metric expresses the expected number of citations per year for an article published in a particular journal, conditioned on the probability that the article will be accepted for publication in that journal. Conditional impact factors may be more appropriate for finding compromise solutions than the raw impact factors because this metric considers the fact that a manuscript that has been rejected accrues zero citations. The three journals with the highest conditional impact factors are the first submission journals for the majority of Pareto-approximate sequences in Fig 3, and *Ecological Monographs* and *Science* are common later options for submission. Of course, pursuing a submission strategy wherein a manuscript rejected from lower-impact factor journals is subsequently submitted to higher-impact factor journals is counterintuitive to most researchers (including the authors), but has been observed by the authors in some cases. One can interpret these results as an indication of the importance of publishing rapidly (fast review times and high acceptance rates) relative to publishing widely (high impact factor). Thus, one heuristic approach for finding submission sequences might be to select from the journals with the highest conditional impact factor, with specific choices made to reflect preferences concerning increased raw impact factors versus increased acceptance rates (for example, opting to begin with *Molecular Ecology Resources* instead of *PLOS ONE* to increase expected citations at the cost of increased review time).

#### Multiple decision-maker rationalities lead to tensions

Consider a multiple author manuscript with a junior author (*T* = 3 years) and a senior author with tenure (*T* = 20 years). The junior author might be a graduate student seeking to publish a chapter from her dissertation, and her top priority is to strengthen her position on the job market. Meanwhile, her advisor may want to bolster the overall impact by publishing in a high-impact factor journal. These different time horizons are an example of multiple decision-maker rationalities [16], which may yield different Pareto-optimal solutions for each author. Our analysis shows, however, that the overall strategies are comparable across researchers. This is due to the similar geometries of the Pareto fronts with respect to the time horizon *T*. These strategies present options to still publish on a relatively short time horizon (no more than five months)–rapidly enough from an author’s job applications perspective–but can still substantially increase the citation count for the senior author relative to an Efficient Strategy (Fig 3C).

Now consider the case of a graduate student who is approaching her Ph.D. defense, with a research focus that is at the forefront of her field that is also quite crowded. This graduate student may assume a relatively high scooping probability *s* = 0.005. In contrast, her tenured advisor may differ in this assessment and, based on his publishing experience, adopts a probability of being scooped of *s* = 0.001. Moreover, the junior author may be more pessimistic about the amount of time required for revisions than the senior author. She assumes that each revision can be expected to take *t*_*R*_ = 45 days, while her advisor assumes *t*_*R*_ = 30 days.

**Fig 5:**
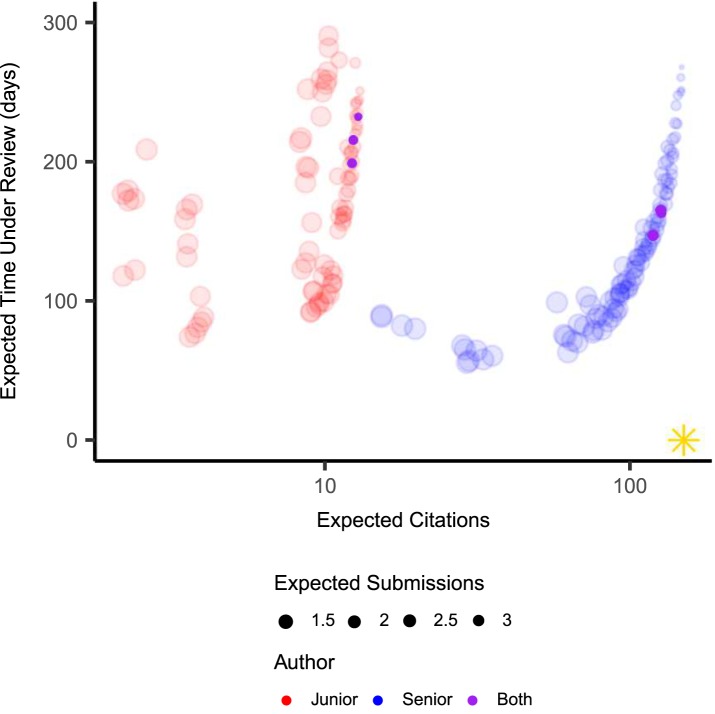
Multiple rationalities can lead to co-author tension. Pareto-approximate solutions for a scooping-averse junior researcher (*T* = 3 years, *s* = 0.005, *t*_*R*_ = 45 days) in red and a less scooping-averse senior researcher (*T* = 20 years, *s* = 0.001, *t*_*R*_ = 30 days) in blue. The *x*-axis (expected citations) is plotted on a log scale to highlight the different ranges of expected citations. The gold star in the lower-right corner indicates the target direction for the displayed objectives.

The higher perceived probability of scooping (and thus accumulating zero citations) compresses her range of possible citation counts (Fig 5); the maximum expected citation rate for the junior author is reduced from 6.4 citations per year (Table 1) to about 4.3 citations per year (13 citations total). Thus, while the junior author could still be aggressive about citation-seeking, the resulting Citation-Maximizing sequences carry with them a high perceived risk (of scooping or long review times) with a relatively low increase in reward (citations per year) compared to sequences that might be classified as Efficient or Compromise solutions. For example, using the review time cutoffs applied previously, the maximum expected citations available under an Efficient solution (no more than 100 days under review) is about 10, while under a Compromise solution (no more than 150 days under review), the maximum expected citations is only slightly higher (10.7).

These different rationalities require discussion and compromise between the two decision-makers, so that the senior author’s desire for maximum exposure can be reconciled with the junior author’s aversion to being scooped. One approach is to look for common Pareto-approximate submission sequences, though these may not always exist, requiring deviation from the approximate Pareto front for one or more decision-makers. In this case, three sequences are non-dominated for both researchers. These sequences and the values for each objective for each researcher are given in Table 2, and are depicted in purple in Fig 5. The shared sequences are generally similar, with the first two journals being some combination of *Ecology Letters* and *Molecular Ecology Resources*, the third journal being *Ecological Monographs*, and *PLOS ONE* occurring towards the end.

**Table 2:** Solutions that are Pareto-approximate for two authors.

Submission sequence	Expected citations		Expected submissions		Expected time under review	
	Junior author	Senior author	Junior author	Senior author	Junior author	Senior author
1. *Ecol Lett* 2. *Mol Ecol Resour* 3. *Ecol Monogr* 4. *PLOS ONE* 5. *Biotropica*	4.3 y^-1^	6.4 y^-1^	3.7	3.1	233 days	164 days
1. *Mol Ecol Resour* 2. *Ecol Lett* 3. *Ecol Monogr* 4. *ISME J* 5. *PLOS ONE*	4.2 y^-1^	6.4 y^-1^	3.3	3.0	216 days	166 days
1. *Mol Ecol Resour* 2. *Ecol Lett* 3. *Ecol Monogr* 4. *PLOS ONE* 5. *Biotropica*	4.1 y^-1^	6.0 y^-1^	3.2	2.9	200 days	148 days

These solutions come close to maximizing the expected citations for the junior researcher, while the senior author can expect more than 75% of the maximum citations he could obtain (indeed, the third sequence in Table 1 is classified as a Compromise solution by our previous analysis). The junior author’s concern about revision time and scooping leads to longer expected review times for her. Thus, from her perspective, these solutions may not be as ideal as solutions with fewer expected citations but substantially less time under review and fewer submissions. While the senior author may be reasonably confident of success in publishing the manuscript in one of the journals (the acceptance probability from his viewpoint is over 81% for all three common solutions), the junior author may be more concerned about receiving five rejections (the maximum acceptance probability from her perspective is about 58%). However, these solutions can serve as a starting point for negotiations between the two collaborators.

From the perspective of the conditional impact factor heuristic described earlier, the solutions presented in Table 2 follow a similar ordering. The first three journals consist of three of the top four (excepting *PLOS ONE*) conditional impact factors, and *PLOS ONE* is included as the fourth or fifth journal in each of these compromise sequences. The positioning of *Molecular Ecology Resources* over *Ecology Letters* for two of the three sequences despite the latter’s higher conditional impact factor reflects the decreased aggression in citation-seeking required due to the junior author’s risk-averse preferences regarding revision time and scooping probability. If the junior author were more optimistic, we would expect to see *Ecology Letters* appear first more frequently.

It may not always be the case that these compromise solutions occur across all of the collaborators’ Pareto-approximate sets, particularly as the number of coauthors increases. It may also be the case that the compromise solutions still perform relatively poorly for certain co-authors (for example, the junior author in our example may not be satisfied with overall acceptance probabilities below 60%). In this case, methods such as the Analytic Hierarchy Process [28] may be used to formulate new objectives that combine the preferences of each stakeholder. Another approach is to combine an MOEA optimization for a “typical” set of parameter values with methods from robust decision-making to explore the robustness of sequences to differing combinations of parameter values. The combination of multi-objective optimization and robustness analysis forms the so-called Multi-Objective Robust Decision-Making (MORDM) framework (17,23,25).

### Discussion and caveats

The analyses of submission decision problem, as the objectives are formulated here and in ref. [10], are adorned with many caveats. For example, we assume that all manuscripts have equal probability of acceptance at a given journal. Others have parameterized the acceptance probability as a function of the manuscript quality [29]. This is arguably more realistic, but requires a subjective judgment that will differ for each manuscript. Due to the subjectivity of assessing manuscript quality, it may be more appropriate to treat this as an uncertain parameter in a robustness analysis, as described earlier. Additionally, not all manuscripts are appropriate for all publication outlets. The considerations of manuscript quality and content could be incorporated by reducing the pool of journals to only those in which the manuscript in question could reasonably be published [10]. Different co-author values for manuscript quality could also be factored into this analysis through author-specific scale factors (between 0 and 1) that modulate the expected acceptance rate in Eqs (2), (3) and (5). An optimistic author would set this scale factor to one and a pessimistic author would set this factor less than one.

We do not intend to explicitly recommend that a researcher should follow some of the Pareto-approximate sequences (e.g., from Tables 1 and 2). For example, some of the citation-maximizing sequences submit to higher-impact factor journals after submitting to lower-impact factor journals, which may seem counterintuitive to some researchers. Indeed, we have observed that the Pareto-approximate sequences, even those that are aimed at maximizing expected citations, tend to be ordered more by conditional impact factors than the raw journal impact factors. Furthermore, the impact factor, acceptance rate, and time-to-decision data set is from 2012 and is specific to Ecology [10], so decisions regarding contemporary strategies for journal submission require consideration of the *characteristics* of the journals along the pathways recommended here. Additionally, researchers may decide to submit their manuscripts to journals in a sequence based on qualitative considerations such as prestige [11], even though these sequences may appear sub-optimal with regard to the quantitative metrics employed here. While some of these considerations may be quantified and incorporated into the optimization procedure, researchers will likely need to make subjective judgments regarding other factors after optimization.

The results presented here are intended to illustrate a framework for understanding the trade-offs and the balancing of competing decision-makers’ interests that are inherent in the journal submission decision problem. These methods show how a general understanding of the trade-offs can provide insight into non-obvious ways to generate journal submission sequences that balance researchers’ values (such as in Table 2), and illustrate the value in *a posteriori* decision-making procedures such as those used in this work. Generating submission sequences tailored to the preferences of a specific set of co-authors likely requires manual inspection of the Pareto-approximate solutions presented here, even within a strategy set consistent with the co-authors’ values. For example, the maximum-citation sequences in Table 1 begin with *Ecology Letters* (impact factor 18) and proceed to *Science* (impact factor 31), if rejected. This counter-intuitive behavior is driven by the fact that the time-to-decision for *Science* in the data set is 84 days, with a low acceptance probability of 7%. On the other hand, the time-to-decision for *Ecology Letters* is 33 days with a 12.4% probability of acceptance [10]. A “desk rejection” by the editor at *Science*, however, is considerably faster (about one week). Finally, our study–as well as ref. [10]–is silent on the question of whose time is allocated to making the revisions (*t*_*R*_) and whose efforts go into the resubmissions. These can be important considerations when honing submission sequences to decision-makers’ particular goals.

The analysis presented here is a limited version of the robust decision-making analysis present in the full MORDM framework (16,19,21). Under this analytic framework, after finding the approximate Pareto front for expected values of uncertain parameter values (such as revision time and scooping probability), selected solutions are tested against a variety of states of the world to determine under what combinations of parameters they perform acceptably well or unacceptably poorly. This full analysis, which is beyond the scope of this didactic example, can more fully inform decision-makers about the risks they may face if they have made incorrect assumptions about the “true” parameter values [20]. The authors intend to illustrate this analytic method in a subsequent work.

## Conclusions

We use a multi-objective optimization analysis to explicitly illustrate the trade-offs inherent in deciding on which journal(s) to send a manuscript. Our results expand upon previous work by representing the tension brought on by multiple rationalities surrounding co-author values such as time horizon and perceived probability of being scooped. This analysis serves as an introduction to the more rigorous robust decision-making framework which often accompanies this method of decision analysis. We implement an open source and freely available multi-objective optimization framework and demonstrate the relative efficiency of our approach over that of previous work (Fig 2). We show that optimal solutions may be partitioned into different classes of strategies, though here we focus on three, which we call Efficient, Citation-Maximizing and Compromise. Citation-Maximizing Strategies are attractive for senior researchers but may be less suitable to junior researcher. This is because the junior researcher’s time horizon is not sufficiently long to accrue a substantially larger number of citations as compared to an Efficient or Compromise Strategy. Furthermore, Citation-Maximizing Strategies also require more time under review and carry a higher risk of rejection from all journals. We find that the conditional impact factor is a useful heuristic metric to generate Compromise and Efficient submission sequences. Moreover, we find that this method of analysis can inform multiple researchers with different interests, assumptions, and perspectives and can help find starting points for negotiations about submission strategies.

## Supporting information

S1 Fig Parallel axis plot including all Pareto-approximate submission sequences. (EPS)

S2 Fig Pareto-approximate submission sequences excluding *PLOS ONE* from the analysis. (EPS)

S3 Fig Parallel axis plots for the three submission strategies, excluding *PLOS ONE* from the analysis. (EPS)

S1 Text Derivation of the mathematical formulation of the submission decision problem. (PDF)
