# Supplementary material for: A multi-objective decision-making approach to the journal submission problem
Source: PLoS One. 2017 Jun 5;12(6):e0178874. doi: 10.1371/journal.pone.0178874 (PMC5459441; doi:10.1371/journal.pone.0178874)
Supplement: S1 Text — (PDF) [file pone.0178874.s004.pdf]

S1 Text

Accompanying “A Multi-Objective Decision-Making Approach to the Journal Submission Problem”

Authors: Wong et al.

Corresponding author: twong@psu.edu

Model Derivation

Probability of manuscript acceptance

Let T represent the time horizon over which the researcher wishes to maximize citations. Let an enumeration of journals be indexed by j, and suppose the authors of a manuscript only intend to submit to N journals total. Suppose the authors intend to submit the manuscript, in order, to journal j=1, 2, …, N. Let α_j, λ_j, and τ_j denote the acceptance rate, expected number of citations for an article in the journal over the course of a year (which we approximate using the journal impact factor), and the expected time from submission to publication for journal j, respectively. Let s denote the probability (per day) of an article being scooped and t_R denote the time (in days) required to make revisions and resubmit a manuscript. We adopt the assumption that a manuscript that has been “scooped” is essentially worthless and receives no citations, and that the probability of being scooped is constant in time (1).

For each journal $j=j^*$, acceptance at journal j^* is conditioned on rejection at journals j=1, 2, …, j^*-1. The probability of rejection at journal $j=k$ is 1- α_k, so the probability of rejection at each of these journals is given by $\prod_{k=1}^{j^*-1}(1-\alpha_k)$. Acceptance at journal j^* is also conditioned on the manuscript not being scooped during the time between rejection from journal j=1 and decision from journal j=2 (1-$s^{\tau_1+t_R}$), not being scooped during the time between rejection from journal j=2 and decision from journal j=3 (1-$s^{\tau_2+t_R}$), and so on to the time between rejection from journal j^*-1 and acceptance at journal j^* (1-$s^{\tau_{j^*-1}+t_R}$). Thus, the probability of rejection at each of journals j=1, 2, …, j^*-1 and the manuscript not being scooped anywhere along the way is

$$p_{j^*} = \prod_{k=1}^{j^*\text{-}1}(1\text{-}\alpha_k)(1\text{-}s)^{\tau_k+t_R}. \quad \text{(S1)}$$

The probability of acceptance at journal j^* is the acceptance rate for journal j^* (α_k), conditioned on the probability of rejection at each of journals j=1, 2, …, j^*-1 and the manuscript not being scooped anywhere along the way – that is, conditioned on p_{j^*}. So the required conditional probability of acceptance at journal j^* is

$$p_{a,j^*} = \alpha_{j^*}\prod_{k=1}^{j^*\text{-}1}(1\text{-}\alpha_k)(1\text{-}s)^{\tau_k+t_R}. \quad \text{(S2)}$$

For any two journals in the submission sequence, indexed by distinct $i, j \in \{1, 2, …, N\}$, the events of acceptance of the manuscript by journal i and acceptance by journal j are independent (conditioned by rejection at previous journals in the sequence and the manuscript not being scooped). Therefore, the probability of the manuscript being accepted by journal i or being accepted by journal j is $p_{\boldsymbol{a},i} + p_{\boldsymbol{a},j}$. This independence relation holds for all j=1, 2, …, N. Thus, the total probability of acceptance of the manuscript by any journal along the submission sequence indexed by j=1, 2, …, N is

$$p_a = \sum_{j=1}^{N}\alpha_j\prod_{k=1}^{j\text{-}1}(1\text{-}\alpha_k)(1\text{-}s)^{\tau_k+t_R}. \quad \text{(S3)}$$

Eq (S3) gives rise to Eq (1) in the main text, using the fact that $p_a + p_r = 1$.

Expected citation count

Next, we derive Eq (2) in the main text, which gives the expected number of citations for a given time horizon (T), scooping probability (s), time for revisions (t_R), submission sequence (indexed by j=1, 2, …, N), journal impact factors (λ_j), journal expected time to decision (τ_j), and journal expected acceptance rates (α_j). Define by T^* the number of years required for the manuscript to be published in journal j^*. T^* includes the amount of time needed for review at journals j=1, 2, …, j^* as well as revision time in before submission to journals j=2, 3, …, j^*. The amount of time needed for review along the sequence of journals is given by

$$T_{review,j^*} = \sum_{j=1}^{j^*}\tau_j, \quad \text{(S4)}$$

and the amount of time needed for revisions along the sequence of journals is given by

$$T_{revise,j^*} = (j^* - 1)\, t_R. \tag{S5}$$

The total time over which the manuscript will accumulate citations is then

$$T - T_{review,j^*} - T_{revise,j^*} = T - \sum_{j=1}^{j^*} \tau_j - (j^* - 1)\, t_R. \tag{S6}$$

The expected number of citations if the manuscript is accepted in journal j^* is the expected number of citations per year (approximated by the journal impact factor, as described in the main text) multiplied by the time over which citations are accumulated (Eq (S6)). Thus, if the manuscript is published in journal j^*, then the expected number of citations is

$$C_{j^*} = \lambda_{j^*} \left[T - \sum_{j=1}^{j^*} \tau_j - (j^* - 1)\, t_R\right]^+. \tag{S6}$$

In Eq (S6), the "+" superscript implies that negative values of the term in square brackets should be replaced with 0. This accounts for the possibility that the researcher's time horizon ends while the manuscript is still in review. Considering now the full submission sequence j=1, 2, …, N, the expected number of citations is

$$C = \sum_{j=1}^{N} C_j\, p_{a,j}. \tag{S7}$$

Eq (2) in the main text immediately follows from substituting Eqs (S2) and (S6) into Eq (S7), and using the fact that with probability p_r the manuscript is rejected from all journals along the sequence and as a result, receives no citations.

Expected time in review

As in the main text, define j_{max} as the largest index j=1, 2, …, N for which the following expression is true:

$$T - \sum_{i=1}^{j} \tau_i - (j - 1)\, t_R > 0. \tag{S8}$$

The manuscript will receive a decision from each journal j=1, 2, …, j_{max}. The manuscript falls into one of two independent cases: (i) the manuscript is not accepted or (ii) the manuscript is accepted for some journal indexed by $j \leq j_{max} \leq N$.

Case (i) occurs with probability given by $1 - \sum_{j=1}^{j_{max}} p_{a,j}$, where $p_{a,j}$ is calculated as in Eq (S2). In this case, the time in review is interpreted as either rejection by all N journals or expiration of the time horizon before the manuscript could be accepted. In either event, the time necessary to reach final decision on the manuscript is given by the time horizon T if time has expired, or by $\sum_{i=1}^{j_{max}} \tau_i + (j_{max} - 1)\, t_R$ if the time horizon has not expired. The use of the "min(…)" function in Eq (5) in the main text chooses between these two events, and the second term in Eq (5) represents case (i), where the manuscript has not been accepted to any journal:

$$P_{case\ (i)} = \min\left(T, \sum_{i=1}^{j_{max}} \tau_i \text{-} (j_{max}\text{-}1)\, t_R\right) * (1\text{-} \sum_{j=1}^{j_{max}} p_{a,j}). \quad \text{(S9)}$$

Case (ii) consists of j_{max} alternative cases, in which the manuscript is accepted by journal j=1, 2, …, j_{max} with probabilities given by $p_{a,j}$, respectively. Thus, case (ii) occurs with probability given by $\sum_{j=1}^{j_{max}} p_{a,j}$. If the manuscript is accepted by journal $j^* \leq j_{max} \leq N$, then the total amount of time spent in review is necessarily positive and less than T, based on the condition of Eq (S8). The total time spent in review is then given by $\sum_{i=1}^{j^*} \tau_i + (j^* - 1)\, t_R$, with probability given by p_{a,j^*}. The first term of Eq (5) in the main text immediately follows by accumulating these j_{max} alternative outcomes and their probabilities:

$$P_{case\ (ii)} = \sum_{j=1}^{j_{max}} \left[\sum_{i=1}^{j} \tau_i + (j\text{-}1)\, t_R\right] p_{a,j}. \quad \text{(S10)}$$

Finally, Eq (5) of the main text is the sum of the two independent events of case (i) or case (ii), given by Eqs (S9) and (S10):

$$P = P_{case\ (i)} + P_{case\ (ii)}. \quad \text{(S11)}$$

Expected number of submissions

The same two cases as expected time in review apply to determining the expected number of manuscript submissions. In case (i), the manuscript has been rejected from each of the journals indexed by j=1, 2, …, j_{max}. It is possible that the time horizon expired after an additional submission but before a decision by this journal could be reached (to yield a total of j_{max}+1 submissions). However, in the broad majority of cases examined in our study, this was not the case. Additionally, in this situation, because the time horizon has expired, zero citations are

accumulated (Eq (S6)), so the solution will not be found to be Pareto approximate anyhow. Thus, we make the simplifying assumption that j_{max} represents the total number of submissions in case (i), where the manuscript is rejected from all journals to which it was submitted. The expected number of submissions, conditioned on case (i), is then:

$$R_{case\ (i)} = j_{max}{}^{*}(1\text{-}\sum_{j=1}^{j_{max}} p_{a,j}). \tag{S12}$$

In case (ii), the manuscript is accepted by some journal $j^* \leq j_{max} \leq N$ with probability given by p_{a,j^*}. The expected number of submissions, conditioned on the j_{max} alternative outcomes within case (ii), is:

$$R_{case\ (ii)} = \sum_{j=1}^{j_{max}} j^{*} p_{a,j}. \tag{S13}$$

The expected total number of manuscript submissions, given by Eq (3) of the main text, follows from the sum of the two independent events of case (i) (Eq S12) or case (ii) (Eq S13):

$$R = R_{case\ (i)} + R_{case\ (ii)}. \tag{S14}$$

References

1. Salinas S, Munch SB. Where should I send It? Optimizing the submission decision process. PLoS One. 2015;10(1).
